# Different Trajectories of Depressive Symptoms in Children and Adolescents: Predictors and Differences in Girls and Boys

**DOI:** 10.1007/s10964-012-9858-4

**Published:** 2012-11-16

**Authors:** Carolin Fernandez Castelao, Birgit Kröner-Herwig

**Affiliations:** Department of Clinical Psychology and Psychotherapy, Georg-August-University of Göttingen, Goßlerstraße 14, 37073 Göttingen, Germany

**Keywords:** Depressive symptoms, Children and adolescents, Developmental trajectories, Gender differences

## Abstract

The development of depressive symptoms in childhood and adolescence can follow different pathways. This study examined heterogeneity in the development of self-reported depressive symptoms and the predictive influence of mothers’ depressive symptoms, the number of life events, and loss events via growth mixture modeling over a four-year period in a large community sample of German children and adolescents (*N* = 3,902; mean age 11.39 years; 49.6 % female). This procedure was conducted for the total sample as well as for separate samples of girls and boys. Four different classes of trajectories for the total and the girls’ model were identified, but only three classes for the boys. Girls showed higher intercepts and stronger increases in symptoms over time, whereas boys displayed stronger decreases. In the total model, mothers’ depressive symptoms and the number of life events significantly increased the level of depressive symptoms. In the gender models, only mothers’ depressive symptoms showed significant influence on the level of symptoms in girls and boys, whereas for life events this was only true for boys. In every model, the significant predictors discriminated at least between some classes. Loss events showed no significant influence in any model. In sum, there are meaningful differences in the development of depressive symptoms in girls and boys. These results have several implications for prevention and future research.

## Introduction

Depressive symptoms are among the most prevalent mental health problems in childhood and adolescence (Fergusson et al. 2005). Studying the development of symptoms is of great importance because they can result in serious personal suffering as well as in significant public health concerns (Pine et al. 1999). Moreover, a better understanding of their development can help to implement adequate preventive strategies and to reduce possible negative consequences. Many studies have shown that depressive symptoms are stable or even increase through childhood and adolescence (e.g., Mesman and Koot 2000; Pine et al. 1999). A rising trend with age is often found, particularly in the transition from childhood to adolescence. At the age of 12 or 13 years, adolescents suffer more often and more seriously from depressive symptoms than their younger companions (e.g., Angold et al. 2002; Cole et al. 2002). Furthermore and compared to boys, girls at the age of 13 or 14 are two to three times more likely to be afflicted by depressive symptoms and also are affected more seriously by it (e.g., Ge et al. 2001; Hankin 2009; Nolen-Hoeksema and Girgus 1994). While adolescent girls manifest increasing levels of depressive symptoms, boys show significantly less increasing (Cole et al. 2002; Hankin 2009), stable, or decreasing symptoms over time (Burstein et al. 2010; Ge et al. 2001).

Despite various studies, little is known about different *types of* *individual trends* in the development of depressive symptoms. In recent decades, most longitudinal studies have analyzed the *mean trend* of depressive symptoms over time by using latent growth modeling techniques or hierarchical linear modeling (e.g., Burstein et al. 2010; Cole et al. 2002; Garber et al. 2002; Ge et al. 2001; Hankin 2009). These statistical methods illustrate unobserved variability in developmental courses through the allowance of variance in growth factors (random effects). It is often suggested that research should focus on categorical heterogeneity in the development of depressive symptoms rather than assume that all children follow—with some variability—the same course (Hammen et al. 2008). In particular, researchers in developmental psychopathology have emphasized the importance of identifying distinct subgroups of development in depressive symptoms (e.g., Cicchetti and Rogosch 1996). Such an approach should help to uncover important—so far hidden—developmental differences in emotional adjustment during childhood and adolescence. It might thus help to answer important questions for prevention and diagnostics (Dekker et al. 2007; Stoolmiller et al. 2005). First, different pathways of development might be detected, and second, possible different causes for different developmental groups might be identified. Based on the vulnerability-stress model (e.g., Hankin and Abela 2005), it can be assumed that youth, who differ in their vulnerability and/or who encounter stress in different ways during their development may vary in the level and course of depressive mood (Cicchetti and Toth 1995; Rutter 1989). These assumed variations in quantity and quality can be described well by analyzing different subgroups of developmental trajectories.

Advanced statistical approaches in growth modeling offer the opportunity to estimate different groups of individual trajectories. These are growth mixture modeling (GMM; Muthén 2001; Muthén and Muthén 2000) and latent class growth analysis (LCGA; Nagin 1999). Both represent a flexible and up to date statistical procedure for analyzing longitudinal repeated measures data and are useful for representing heterogeneity in developmental trajectories (e.g., Jung and Wickrama 2008; Muthén 2004). In comparison with traditional growth modeling techniques, GMM and LCGA try to measure heterogeneity by estimating two or more different classes of trajectories instead of only capturing differences by assuming variability around one mean trajectory.

So far, four recent studies have analyzed different trajectories of depressive symptoms with GMM or LCGA (Brendgen et al. 2005; Dekker et al. 2007; Rodriguez et al. 2005; Stoolmiller et al. 2005). In a sample of 206 male adolescents, Stoolmiller et al. (2005) found four classes of trajectories with differences in the level as well as the course of depressive symptoms over a period of ten annual assessment points (“very low-stable”, “moderate-decreasing”, “high-decreasing”, and “high-persistent”). Brendgen et al. (2005) analyzed a sample of 414 girls and boys aged 11–14 over 4 years and identified four distinct classes of trajectories with stable or increasing levels of symptoms over time. Furthermore, studying a sample of 925 adolescents from grades 9 to 12 over 5 years, Rodriguez et al. (2005) reported three different groups of trajectories with high, moderate, and low levels of depressive symptoms.

Given the strong evidence for gender differences regarding depressive symptoms, it is worth assuming that there will be differences in the types and prevalence of trajectories for girls and boys. To our knowledge, the study by Dekker et al. (2007) is the only one to compare differences in classes of trajectories of depressive symptoms between girls and boys. They identified six different subgroups per gender in a sample aged 4–18 years (*N* = 2,076) over five waves. Heterogeneity was found not only in the level of symptoms, but also in the shape of the curves. In general, girls displayed higher levels and more increasing or chronic trajectories, whereas the majority of boys showed low levels and more overall decrease.

Dekker et al. (2007) used parental reports in their study and did not use self-reports, although there is evidence that children as well as adolescents and their parents differ widely in their report of depressive symptoms (Cole et al. 2002; Garber et al. 2002). It can be assumed that parents rely more on external symptoms and are often not aware of the inner mental state of their children (Mesman and Koot 2000). Kröner-Herwig et al. (2009) found the parent–child agreement ranked the lowest for depressive symptoms in comparison with a range of other symptoms. These findings underline the importance of self-reports. Cantwell et al. (1997) even pointed out that adolescents are the optimal source of information on depression in adolescents. To our knowledge, there is no published research to date that has conducted an analysis of different developmental classes of depressive symptoms separately for girls and boys based on self-reports, which is assumed to assess emotional processes in a more valid way.

It can be assumed that variations in the course of depressive symptoms depend on different risk factors. On the basis of the vulnerability-stress model, many researchers focused on the role of exposure to stress in their attempt to predict depressive symptoms (e.g., Cole et al. 2006; Hankin et al. 2007). The association between stressors and depression might increase in the transition from childhood to adolescence (Larson and Ham 1993). For our study, we selected three psychosocial stressors because of their significant role in adolescence (e.g., Ge et al. 2001; Rudolph 2002); maternal depressive symptoms, life events, and loss events. Various longitudinal studies have demonstrated that the level or course of maternal depressive symptoms predicted subsequent increases in children’s symptomatology (Garber et al. 2011; Garber and Cole 2010; Kouros and Garber 2010). In addition, Garber et al. (2002) revealed that adolescents whose mothers had depressive symptoms had higher levels of symptoms than their peers whose mothers had no symptomatology. The influence of negative life events also has yielded relevant results. In some prospective studies, it was shown that the experience of life events led to higher levels of depressive symptoms in childhood and adolescence (Ge et al. 2001; Johnson et al. 2012; Nolen-Hoeksema et al. 1992). Studies that focused on different types of life events revealed that events related to the loss of an important person, for example, the death of a parent (Cerel et al. 2006), the divorce of parents (Ge et al. 2006; Rønning et al. 2011), or breaking up with girlfriend or boyfriend (Monroe et al. 1999), had the strongest influence on subsequent depressive symptoms compared with other life events.

The results of past research already have proved the importance of these three variables in the context of depressive symptoms. Despite this well-established general knowledge, little or no information about their ability to predict overall growth in different classes or to discriminate between these different classes is available due to the small number of GMM or LCGA studies. So far, only two studies have addressed these predictive relationships. Stoolmiller et al. (2005) reported that life events had a significant effect on the level and course of different trajectories of depressive symptoms in adolescent boys. Parental depressive symptoms only showed a significant influence on the level of symptoms. Both variables predicted class membership as well. Sterba et al. (2007) yielded comparable results for the impact of maternal postpartum depressive symptoms on trajectories of *internalizing* symptoms. To clarify the importance of these predictors for different developmental classes, further examination is necessary.

Studies about differences in predictive relationships between girls and boys also are required. As Dekker et al. (2007) did not analyze any predictors relating to sex, knowledge of gender differences in the relationships between different subgroups of depressive trajectories and related predictors is scant. However, results based on traditional growth modeling or correlational designs sometimes reveal that gender has an influence on the predictive impact of maternal depressive symptoms, with more adverse effects for girls (Cummings et al. 2005; Foster et al. 2008; Jenkins and Curwen 2008). Furthermore, in their longitudinal study, Ge et al. (1994) found that adverse life events were associated with an increase in depressive symptoms in girls but not in boys.

## Hypotheses

On the basis of previous results, it seems crucial to extend the knowledge of different subgroups in the development of depressive symptoms in childhood and adolescence, especially with regard to possible differences in girls and boys. In doing so, the use of self-reports as well as the inclusion of predictive variables should allow for better insight into varying developmental pathways. The first aim of our study was to examine different classes of trajectories relying on self-reported depressive symptoms over four annual waves in our total sample of children and adolescents. In consideration of previous research, we expected to detect three or four different classes of trajectories in the total sample. A further aim was to analyze whether and to what extent mothers’ depressive symptoms, the number of life events, and loss events as a particular type of life event, can predict the level and rate of change of depressive symptoms as well as class membership. Based on the results of earlier studies, we expected that the variables maternal depressive symptoms and the number of life events would influence significantly the level of children’s depressive symptoms and would be able to discriminate at least between some classes. We did not have any comparable information about predictive relationships for the variable loss events, but we supposed that it would show similar influences. In a second step, we examined the models separately for girls and boys to assess possible gender differences with regard to classes and predictors. Based on existing literature, we assumed that higher intercepts would be found for girls and that those trajectories with a constantly increasing time trend should prevail for girls, whereas they would decrease over time for boys. No particular hypotheses regarding possible differences in predictive relationships for girls and boys were generated, because of the lack of adequate previous research.

## Methods

### Participants and Procedure

The study sample was originally drawn from a large population-based longitudinal epidemiological study on headache and other health variables (e.g., depressive symptoms) in German children and adolescents (age 7–14 years). Children and their parents were selected randomly from four districts in Lower Saxony (Germany) and were informed about the research project via postal survey. The data collection took place during four annual waves. Each year, participants were asked to fill in a parent questionnaire or a child questionnaire, respectively, which included a range of health-related as well as psychosocial items. To avoid excessive demands on them, only the children ≥9 years old were asked to complete the questionnaire. The study was accepted by the Ethics Committee of the German Association of Psychology. Furthermore, data safety procedures were approved by the university data protection representative. For more details of the conduct of the study and the initial data collection, see Kröner-Herwig et al. (2007).

From the 8,800 families originally contacted, a sample of 5,565 families was available after excluding the non-responder (*n* = 3,221) and those families who returned questionnaires with over 50 % missing data (*n* = 14). As we used self-report data in this study, we only included data from those children who were ≥9 years old at baseline (*N* = 4,045) in our analysis. The sample size was reduced again by only including participants with valid data on depressive symptoms for at least the first measurement (*N* = 3,902; mean age 11.39 years [range 9–14 years]; girls 49.6 %). Upper and middle socioeconomic status (SES) was overrepresented (45.6 % each). However, our study sample was comparable to the original sample with regard to other demographic variables (e.g., gender and age). Half (51 %; *n* = 1,972) of the participants provided valid data on depressive symptoms at all four waves, the remainder of the participants provided data at three or less measurements (*M* = 2.99, *SD* = 1.19).

### Measures

#### Depressive Symptoms

Depressive symptoms in children and adolescents were assessed according to the Youth Self Report depression scale (YSR; Achenbach 1991). Not all items of the original scale were adopted due to the complexity and length of the complete questionnaire in our study: we selected eight items because they showed strong item-scale correlations in the original version of the YSR (Kröner-Herwig et al. 2008). This reduced scale correlates well with the comprehensive scale (*r* > 0.80; see Kröner-Herwig et al. 2008). The internal consistencies in all four surveys were satisfactory (α = 0.83 to α = 0.85). The range of the response scale was altered from a three-point- to a five-point scale (1 = *never* to 5 = *always*) to allow for more differentiated results and to adapt it to most of the other scales of our questionnaire. The children answered eight questions with regard to the following symptoms during the last 3 months: unhappy/sad, worries, feel not loved, nervous/tense, worthless/inferior, fearful/anxious, be suspicious, and feel guilty. These eight items were merged into a total mean score of depressive symptoms. Existing literature confirms the strong overlap of depressive and anxiety symptoms (e.g., Angold et al. 1999; Axelson and Birmaher 2001). Anxiety is a well-known component or correlate of depressive mood, especially in childhood and adolescence (Essau and Petermann 1999). For these reasons as well as for the sake of terminological simplicity, we label the construct depressive symptoms. Depressive symptoms were assessed at each of the four measurements.

#### Mothers’ Depressive Symptoms

Information about mothers’ depressive symptoms was drawn out of the parents’ questionnaire in the first wave. The items were derived from the parental mental health scale of the German version of the Child Health Questionnaire (CHQ-PF50; Landgraf et al. 1998). The CHQ measures the physical and psychosocial health status and well-being of children and adolescents and also the well-being of parents. Parents answered the following five items related to the last 4 weeks (five-point scale; 1 = *never* to 5 = *always*): feel nervous, feel depressed, feel composed, feel sad and discouraged, and feel happy. For the analyses of predictive influences on growth and class membership, the mean score of depressive symptoms was used. The items “composed” and “happy” were recoded so that higher scores indicated a higher level of symptoms. The psychometric properties of the CHQ and its subscales had been shown by Landgraf et al. (1998). As we were interested in the specific predictive influence of maternal depressive symptoms, data from this variable was included only if the mother completed the questionnaire (*n* = 3,475). Participants for whom the father or other care-giving persons filled in the parent questionnaire were excluded.

#### Life Events

Information about all life events was taken from the parent questionnaire in the first survey. The items were extracted from the Mannheimer Parent Interview (MEI; Esser et al. 1989). Parents reported on the occurrence of any of the following nine life events within the last 5 years: placement in a boarding school or children’s home, extraordinary change of school, separation from/loss of an important attachment figure (e.g., death, divorce, moving), new person in the family (e.g., birth of sibling, new partner of a parent), chronic illness in the family, serious accident in the family, family under heavy financial burden, nursing of a family member, or hospitalization of the child. For the purpose of analysis, the sum of eight out of nine life events was computed (the number of life events). The life event “separation from/loss of an important attachment figure” (loss events) was analyzed separately regarding the prediction of growth and class membership. Rates of missing data were low (life events: *n* = 3,896; loss events: *n* = 3,833). Due to the quality of the life event assessment, it is not possible to state any information on internal consistency (Streiner 2003), but content as well as face validity can be assumed (Esser et al. 1989).

### Statistical Analysis

GMM was conducted using Mplus 6.1 software (Muthén and Muthén 1998–2010). For model selection, we refer to the Bayesian Information Criterion (*BIC*; Muthén 2001; Nagin 1999; Tofighi and Enders 2008), an information-based index of relative model fit, in which lower values indicate better model fit. The Lo-Mendell-Rubin likelihood ratio-based test of model fit (LMR-LRT) and the more recent parametric bootstrapped likelihood ratio test (BLRT) can be used to compare the absolute fit of a *k*-class model with a *k*-1 class model (e.g., Muthén 2004; Nylund et al. 2007; Tofighi and Enders 2008). Classification accuracy can be considered through the entropy value and the average posterior probabilities for each participant’s most likely class membership (Jung and Wickrama 2008; Muthén and Muthén 2000). In addition, predictive influences on growth factors (intercept and slope) as well as on class membership (latent class variable) can be analyzed via linear or multinomial logistic regression analysis, respectively.

If variances of growth parameters do not differ substantially across classes, these variances can be held equal to reduce model complexity and computational effort (Jung and Wickrama 2008; Tofighi and Enders 2008). For reasons of model convergence, small and insignificant variances of the intercepts can be constrained to zero to achieve a positive model solution (Muthén and Muthén 1998–2010; Nagin 1999). In our analyses, we constrained the variances of the slope factors to be equal across classes and that of the intercepts to be zero. The models were estimated with 500 random sets of start values to find the true maximum likelihood solution and to avoid local solutions.

Mplus uses the full information maximum likelihood (FIML) approach, which allows the inclusion of participants with missing data at *k*-1 assessment periods in the dependent variable. Because the FIML estimation is not possible for missing data in predictors (Muthén and Muthén 1998–2010), the regression analyses are based on reduced sample sizes (see Table 4). Children and adolescents with missing data on predictors did not differ significantly from those with complete data with regard to depressive symptoms at every time, SES, gender, and age (all *p* > 0.05). The minimum covariance coverage was 0.54 which allowed for an adequate estimation.

## Results

### Time and Gender Differences

On average, the children and adolescents in our study showed low to moderate^1^ levels of depressive symptoms with a linear increase over the four waves (*F*_(3,1969_) = 80.13, *p* < 0.001; see Table 1). Girls and boys differed significantly in their levels of depressive symptoms at all four assessment points, but no significant differences were identified in the variables maternal depressive symptoms, the number of life events, and loss events between girls and boys (see Table 1). Correlations between depressive symptoms at every time and all other variables were significant (between *p* < 0.001 and *p* = 0.039) with low to moderate sizes (between *r* = 0.04 and *r* = 0.48). Correlations between gender and predictors did not reach significance.

**Table 1:** Descriptive statistics of dependent variables and predictors

	Total		Girls		Boys		
Variable	*N*	*M (SD)*	*N*	*M (SD)*	*N*	*M (SD)*	
DEP 1	3,902	1.66 (0.58)	1,937	1.75 (0.62)	1,965	1.57 (0.53)	*F*_(1,3900)_ = 93.41***
DEP 2	2,822	1.70 (0.57)	1,442	1.80 (0.61)	1,380	1.58 (0.50)	*F*_(1,2820)_ = 110.77***
DEP 3	2,547	1.80 (0.60)	1,295	1.93 (0.62)	1,252	1.66 (0.54)	*F*_(1,2545)_ = 141.56***
DEP 4	2,378	1.82 (0.61)	1,227	1.98 (0.64)	1,151	1.65 (0.52)	*F*_(1,2376)_ = 193.23***
MDS	3,475	2.44 (0.69)	1,724	2.43 (0.69)	1,751	2.45 (0.69)	*F*_(1,3473)_ = 0.99
LE	3,896	0.82 (0.99)	1,932	0.77 (0.96)	1,964	0.84 (1.02)	*F*_(1,3894)_ = 4.26
LOSS	3,833	0.24 (0.43)	1,895	0.24 (0.43)	1,938	0.23 (0.42)	*F*_(1,3831)_ = 0.38

Differences between girls and boys are shown in the last column

*DEP* depressive symptoms (at different time points), *MDS* mothers’ depressive symptoms, *LE* number of life events, *LOSS* loss events

*** *p* ≤ 0.001

### Growth Mixture Modeling

#### Unconditional Model for the Total Sample

We conducted five GMMs without predictors at the first step to analyze if there were different classes of trajectories of depressive symptoms. Starting with a one-class model, the addition of a further class yielded an increasingly better model fit up to the allowance of four groups (*BIC* = 17201.481, *BIC* = 16649.623, *BIC* = 16483.195, and *BIC* = 16343.160, respectively), but with the assumption of a fifth class the *BIC* started to increase (*BIC* = 16350.390). The result of the LMR LRT supported this finding conclusively in preferring the four-class solution over a three-class solution (*p* = 0.003), whereas the five-class model was rejected (*p* = 0.245). The BLRT confirmed the four-class model (*p* = 0.000). Classification accuracy of the four-group model was good (entropy = 0.82) with average posterior class probabilities between 0.84 and 0.93.

Regarding the four classes of trajectories, the one with the most members (62.5 %) demonstrated a low intercept and a significant upward trend (“low-increasing” class; Table 2). The second largest group, consisting of 28.3 %, showed a slightly increasing course at a moderate level of depressive symptoms (“moderate-slightly increasing”). A further class (8.1 %) started high, but followed a decreasing trend (“high-decreasing”). The smallest class (1.1 %) began with an even higher intercept, but showed a non-significant decreasing process (“very high-stable”). The classes differed significantly with regard to gender and predictors (see Table 3).

**Table 2:** Parameter estimates of unconditional models for the total, girls’, and boys’ sample

Class (class sizes in %)	Intercept^a^ (*SE*)	Slope^b^ (*SE*)
Total		
1. Very high-stable (1.1 %)	3.51*** (0.14)	−0.13 ns (0.10)
2. High-decreasing (8.1 %)	2.75*** (0.06)	−0.12*** (0.02)
3. Moderate-slightly increasing (28.3 %)	2.01*** (0.03)	0.03* (0.01)
4. Low-increasing (62.5 %)	1.32*** (0.01)	0.10*** (0.01)
Girls		
1. Very high-stable (1.3 %)	3.62*** (0.21)	−0.12 ns (0.11)
2. High-slightly decreasing (9.7 %)	2.82*** (0.08)	−0.09*** (0.03)
3. Moderate-slightly increasing (32.7 %)	2.06*** (0.03)	0.05** (0.02)
4. Low-increasing (56.3 %)	1.33*** (0.01)	0.14*** (0.01)
Boys		
1. High-strong decreasing (4.8 %)	2.89*** (0.10)	−0.22*** (0.05)
2. Moderate-stable (25.6 %)	2.00*** (0.05)	−0.01 ns (0.02)
3. Low-slightly increasing (69.6 %)	1.32*** (0.01)	0.07*** (0.01)

* *p* ≤ 0.05, ** *p* ≤ 0.01, *** *p* ≤ 0.001

^a^Variances of intercepts were fixed at zero

^b^Variances of slope factors were constrained to be equal across classes (total: 0.021; girls: 0.024: boys: 0.015) and were all significant (*p* ≤ 0.001)

**Table 3:** Characteristics of the classes of trajectories in the total, girls’, and boys’ model

	Mean by class (*SD*)				
Covariate	1	2	3	4	
Total	Very high-stable	High-decreasing	Mod.-slightly increasing	Low-increasing	
MDS	2.76 (0.80)	2.64 (0.67)	2.55 (0.68)	2.36 (0.68)	*F*_(3,3471)_ = 29.33***
LE	1.33 (1.25)	1.08 (1.10)	0.88 (1.03)	0.75 (0.95)	*F*_(3,3892)_ = 17.24***
LOSS	0.38 (0.49)	0.32 (0.47)	0.26 (0.44)	0.21 (0.41)	*F*_(3,3829)_ = 8.64***
Gender (% girls)	80.0	67.1	57.7	43.6	*Χ*²_(3)_ = 114.72***
Girls	Very high-stable	High-slightly decreasing	Mod.-slightly increasing	Low-increasing	
MDS	2.67 (0.85)	2.58 (0.67)	2.52 (0.68)	2.34 (0.68)	*F*_(3,1720)_ = 12.65***
LE	1.57 (1.34)	0.99 (1.02)	0.83 (1.03)	0.69 (0.89)	*F*_(3,1928)_ = 11.92***
LOSS	0.55 (0.51)	0.29 (0.46)	0.28 (0.45)	0.21 (0.40)	*F*_(3,1891)_ = 8.55***
Boys	High-strong decreasing	Moderate-stable	Low-slightly increasing	–^a^	
MDS	2.79 (0.66)	2.62 (0.68)	2.37 (0.68)	–^a^	*F*_(2,1748)_ = 30.80***
LE	1.37 (1.21)	0.97 (1.04)	0.79 (0.98)	–^a^	*F*_(2,1961)_ = 18.97***
LOSS	0.35 (0.48)	0.24 (0.43)	0.22 (0.41)	–^a^	*F*_(2,1935)_ = 4.36**

*MDS* mothers’ depressive symptoms, *LE* number of life events, *LOSS* loss events. Gender was coded with 0 = boys and 1 = girls. Differences between classes are shown in the last column

^a^Not defined

** *p* ≤ 0.01, *** *p* ≤ 0.001

#### Conditional Models for the Total Sample

All predictors (mothers’ depressive symptoms, the number of life events, and loss events) were examined simultaneously in the same model to calculate their predictive influences on growth factors and class membership. To justify our separate analyses for girls and boys, we examined the influence of gender in our total model as well. Based on the *BIC* values and the results of the LRM LRTs the four-class solution (*p* = 0.003) was chosen in the conditional model again. This result was supported by the BLRT (*p* = 0.000). The inclusion of predictors did not change the model in a substantial way with regard to classification accuracy, size, shape, and characteristics of each class. Therefore, the results are not presented in detail.

The linear regression revealed significant effects of maternal depressive symptoms and the number of life events on the intercept, indicating that higher values of mothers’ symptoms and life events increased the level of depressive symptoms (see Table 4). Gender had a significant effect on the linear slope, suggesting that being female was associated with a higher increase of symptoms over time. As recommended, the largest class was used as the reference class in multinomial logistic regression analysis on the latent class variable (e.g., Muthén and Muthén 1998–2010). In comparing the “low-increasing” group with the other classes, maternal depressive symptoms increased the relative chance of being in the “moderate-slightly increasing” (*OR* = 1.42) or in the “high-decreasing” (*OR* = 1.53) class. Life events significantly discriminated between all classes and the reference class with the largest odds ratio (*OR* = 1.18) for the differentiation of the “very high-stable” class. Gender showed significant influence on class membership as well. Being female clearly increased the risk of belonging to all other classes with the largest odds ratio (*OR* = 5.49) for the “very high-stable” class. Loss events did not show any significant influence on growth factors or on class membership.

**Table 4:** Influences of covariates in the total, girls’, and boys’ model

		Prediction to		Prediction to class OR (95 % CI)		
Sample	Covariate	Intercept^a^	Slope^**a**^	1 versus 4	2 versus 4	3 versus 4
Total (*n* = 3,422)	MDS	0.06***	0.00	1.49 [0.76–2.85]	**1.53** [1.25–1.87]	**1.42** [1.21–1.67]
	LE	0.02**	–0.01	**1.18** [1.07–1.60]	**1.17** [1.10–1.41]	**1.09** [1.01–1.21]
	LOSS	0.01	–0.00	1.35 [0.61–2.97]	1.27 [0.88–1.85]	1.02 [0.77–1.36]
	Gender	0.01	0.08***	**5.49** [2.15–14.00]	**2.77** [1.95–3.94]	**1.80** [1.43–2.26)
Girls (*n* = 1,693)	MDS	0.04**	0.01	1.34 [0.68–2.65]	**1.42** [1.08–1.87]	**1.39** [1.10–1.75]
	LE	0.02	–0.01	1.25 [0.87–1.80]	1.13 [0.89–1.42]	1.02 [0.86–1.19]
	LOSS	0.04	–0.02	1.98 [0.83–4.70]	1.10 [0.68–1.80]	1.12 [0.78–1.61]
Boys (*n* = 1,729)	MDS	0.07***	–0.01	**1.68** ^b^ [1.20–2.36]	**1.43** ^c^ [1.14–1.80]	–^d^
	LE	0.03*	–0.01	**1.32** ^b^ [1.02–1.70]	0.99^c^ [0.84–1.17]	–^d^
	LOSS	–0.03	0.01	1.32^b^ [0.68–2.56]	1.00^c^ [0.63-1.60]	–^d^

*MDS* mothers’ depressive symptoms, *LE* number of life events, *LOSS* loss events. Gender was coded with 0 = boys and 1 = girls. Significant values are bold

^a^Values in columns indicate linear regression coefficients

^b^1 versus 3

^c^2 versus 3

^d^Not defined

* *p* ≤ 0.05, ** *p* ≤ 0.01, *** *p* ≤ 0.001

#### Unconditional Models for Girls and Boys

**Fig. 1:**
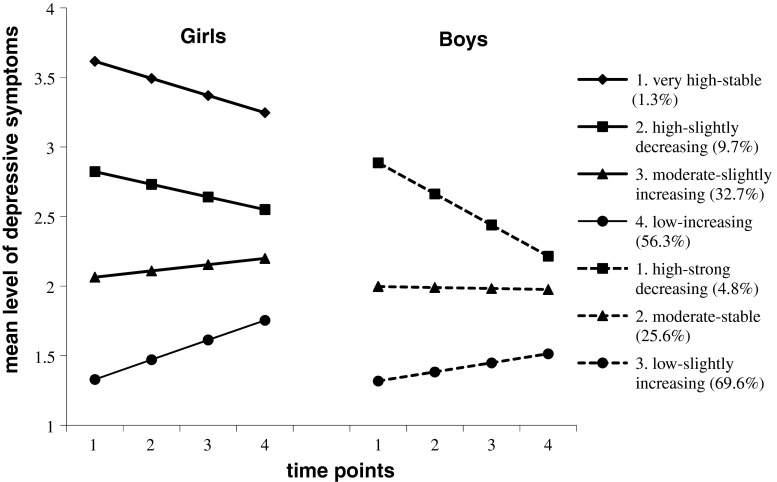
Trajectories of extracted classes for girls (*bold-face lines*) and boys (*dashed lines*)

The separate analysis of genders revealed the following results: The *BIC* values decreased with the addition of further classes until four classes in girls (10272.318, 9280.804, 8977.068, 8932.197, and, 8947.110, respectively) and three classes in boys (8261.596, 7471.330, 7279.717, 7295.659, and 7311.287, respectively) were assumed. The LMR LRT supported these results and rejected the five-class model in girls (*p* = 0.06) and the four-class model in boys (*p* = 0.29), respectively. Hence a four-class solution for girls and a three-class solution for boys were chosen (see Fig. 1). The results of the BLRTs confirmed our model selections (*p*s = 0.000). Entropy in these models was 0.82 in girls and 0.80 in boys with average posterior class probabilities ranging from 0.85 to 0.93 and from 0.84 to 0.94, respectively.

For girls, the largest class, comprising 56.3 % of the sample, displayed a low symptom level and a significant increase over time (“low-increasing”). The second largest class demonstrated a slight upward trend at a moderate level (“moderate-slightly increasing”; 32.7 %). A further class started high but made a slight decrease (“high-slightly decreasing”; 9.7 %) and finally a “very high-stable” class with a non-significant decrease (1.3 %) was identified (see Table 2). In the boys’ model, most participants (69.6 %) belonged to the class with depressive symptoms at a low level and only small increase (“low-slightly increasing”). Furthermore, there was one group with symptoms at a consistently moderate level (“moderate-stable”; 25.6 %) as well as one class which started at a high level of symptoms but experienced a strong decrease (“high-strong decreasing”; 4.8 %). The differences between classes with regard to predictors were significant in both genders (see Table 3).

#### Conditional Models for Girls and Boys

Including the three predictors in one model did not change the models in a substantial way. The *BIC* values as well as the results of the LRM LRTs favored the four-class model for girls (*p* = 0.02) and the three-class solution for boys (*p* = 0.005), respectively. The BLRTs supported these results (*p*s = 0.000).

As can be seen in Table 4, linear regression analysis revealed a significant predictive influence of mothers’ depressive symptoms on the level of depressive symptoms in both genders. The number of life events significantly increased the level of symptoms only in boys. Loss events did not have any significant influence on the level or course of depressive symptoms. Multinomial logistic regression analysis revealed that maternal depressive symptoms significantly predicted class membership for the “high-slightly decreasing” (*OR* = 1.42) and the “moderate-slightly increasing” (*OR* = 1.39) class over the “low-increasing” class in girls and for all classes in comparison with the reference class in boys (largest *OR* [1.68] with regard to the “high-strong decreasing” class; see Table 4). The influence of life events differed with regard to gender. In boys, a higher number of life events increased the probability of being in the “high-strong decreasing” class (*OR* = 1.32) instead of membership in the “low-slightly increasing” class. In girls, the number of life events showed no significant influence in the discrimination of different classes. Loss events did not predict class membership in girls or in boys.

## Discussion

Depressive symptoms are one of the most common mental health problems in children and adolescents (e.g., Fergusson et al. 2005; Rohde et al. 2009). Past research has demonstrated that the mean level of depressive mood increases during the transition from childhood to adolescence, especially for girls (Cole et al. 2002; Ge et al. 2001). However, the assumption of one developmental course does not seem adequate for all young people (e.g., Hammen et al. 2008). The use of novel statistical procedures allows for the examination of different trajectory subgroups and thus permits the detection of different developmental types and their predictors. Within the small group of researchers who applied this method in the context of depressive symptoms, only one study has modeled different trajectory classes for girls and boys separately (Dekker et al. 2007). Hence, the current study is the second to focus on gender differences in subgroups of depressive symptoms. In doing so, we have extended the scope of earlier results in several ways. First, we used self-reports of depressive symptoms, while in most studies ratings of parents were used. Furthermore, we not only assessed different trajectories but also analyzed differences in the predictive relationships between three psychosocial variables (maternal depressive symptoms, the number of life events, and loss events) and growth as well as class membership. To date, no research has examined whether the prediction of class membership differs in girls and boys. By using GMM, we examined the number and types of depressive symptom trajectories and related predictors for the total sample as well as for separate samples of girls and boys.

### Trajectories and Predictors in the Total Sample

As assumed, we found differing time courses in the total sample with a model of four different classes of trajectories best representing the individual courses. The subgroups differed in their mean level, course of symptoms, and percentage of members. The most pervasive pattern in our study was the “low-increasing” class that included almost two- thirds of the sample, followed by almost 30 % in the “moderate-slightly increasing” class. Hence, the large majority of children (>90 %) showed low to moderate levels of depressive symptoms, whereas about 10 % demonstrated a high symptom level, including the “high-decreasing” and the “very high-stable” class. In these two classes, the depressive symptoms never reached a moderate level.

The number of classes we extracted is in line with the results of Brendgen et al. (2005) who also analyzed self-report data of adolescents, even though their sample was smaller and younger than ours. However, they only found classes with stable or increasing trajectories over time, whereas we identified a subgroup with decreasing courses as well. This difference could be explained by differences in age, because for mid-adolescents decreases in symptoms are more likely than for pre-adolescents, who tend to show increases in depressive mood. Stoolmiller et al. (2005) who studied male adolescents and young adults identified decreasing courses as well. The high prevalence of low or moderate levels of depressive symptoms in children and adolescents found in our study is comparable to the results of other studies (Brendgen et al. 2005; Rodriguez et al. 2005).

Predictive relationships with growth factors or class membership can provide additional evidence for meaningful differences between the extracted classes (Muthén 2004). As hypothesized, a higher level of mothers’ depressive symptoms and a higher number of life events were both associated with a higher initial level of depressive symptoms in children and adolescents. As expected, both variables significantly predicted class membership as well, but differed in the number of classes that were distinguished. Interestingly, maternal depressive symptoms did not discriminate the “very high-stable” class from the reference class. Perhaps the power to detect a significant influence was too small because the number of children in that class with data on maternal symptoms was very small (*n* = 19) or the prediction of a very high level of symptoms is subject to other predictors (e.g., life events).

Our results support the findings of earlier studies that also documented comparable predictive influences of life events and depressive symptoms of the mother (Sterba et al. 2007; Stoolmiller et al. 2005). Because these studies were based on different sample sizes and age ranges, as well as on different sets of predictors, the influences of both variables seem to be reliable and robust. With this, they demonstrate their importance in the attempt to predict the severity of depressive symptoms as well as the most likely type of trajectory. Both psychosocial influences are usually highly demanding for children and adolescents because they can trigger a large amount of stress themselves or can even cause related stressful experiences (Cummings et al. 2005).

So far, the influence of loss events has not been studied in the context of different classes of developmental trajectories. Our study was the first to try to close this gap. Our expectation with regard to loss events was not confirmed. The experience of loss events neither allowed for the prediction of class membership nor for the prediction of growth factors. Considering possible reasons for our result, the choice of loss events should be taken into account. We only included the separation from/loss of an important attachment figure in our study. Earlier longitudinal studies have demonstrated significant predictive influence of these types of loss events (e.g., Ge et al. 2006; Rønning et al. 2011). However, other loss events perhaps would have been more specific or more common for our age group, for example, a break up with best friend or—for adolescents—the ending of a romantic relationship (Monroe et al. 1999). One hint underlining this assumption is the very small percentage of children and adolescents who had experienced a loss event in this study. In future studies, the use of more salient events may further clarify the influence of loss events in the prediction of different subgroups.

To provide a basis for our separate analyses for girls and boys, we also analyzed the influence of gender in our total model. Being female was directly associated with a greater increase in symptoms over time, while girls also had an increased relative risk of being in the moderate- and high-level classes. The highest risk (by 5.49 times) was identified for membership in the “very high-stable” class. Similar results were demonstrated by Brendgen et al. (2005) and Rodriguez et al. (2005). These results underline the importance of gender besides that of time-varying influences in the understanding of the development of depressive symptoms and thus justify our separate models for girls and boys.

### Trajectories and Predictors in the Girls’ and in the Boys’ Samples

The gender differences found in the total sample were confirmed and expanded in the separate models for girls and boys. One difference between genders became evident regarding the number of extracted classes, with four classes found for girls but only three for boys. This is a somewhat unexpected finding since Dekker et al. (2007), Sterba et al. (2007), and Toumbourou et al. (2011) found the same number of classes for girls and boys when studying the developments of depressive or internalizing symptoms, respectively. Two of them even found six classes per gender. These discrepancies could be explained by differences in sample size and age, time period, source of information, and statistical procedure. Considering qualitative differences in the types of classes, girls showed higher levels than boys and an increase of depressive symptoms over time double that of boys. Besides, only in girls was a very high level of symptoms found in a small subgroup. Although a decline from a high level of symptoms was identified in both genders, the rate of decrease for boys was more than double that for girls. While a subgroup of boys demonstrated a stable course over time at a moderate level, girls did so at a very high level. As in the total model, in both genders the large majority of children were in the low- and moderate-level classes (girls 89 %; boys 95.2 %). Only a very small subgroup of boys showed trajectories with a high initial level of symptoms (4.8 %), whereas in the sample of girls more than twice as many children demonstrated courses starting at a high or a very high level (11 %). The distributions of membership in low- and high-level classes and the gender-specific characteristics with regard to level and shape of our samples are in line with earlier results (Dekker et al. 2007; Sterba et al. 2007). Moreover, our results fit with the majority of findings with regard to the mean trajectory of depressive symptoms over time, which showed higher levels of symptoms and worse longitudinal courses for girls (e.g., Cole et al. 2002; Garber et al. 2002; Hankin 2009).

Our findings indicate that there is more heterogeneity as well as more severity in the development of depressive symptoms over time for girls than for boys. Unfortunately, the results we derived from the predictive analyses did not provide any adequate explanation for these differences. As in the total model, loss events failed to show any significant influence on growth factors or on class membership. The same reasons as mentioned in the total model section can be assumed for this result. Although there were no significant differences between girls and boys in the occurrence of predictors, girls and boys obviously differed in the type and size of predictive relationships with regard to maternal depressive symptoms and the number of life events. In the sample of boys, both variables, mothers’ depressive symptoms *and* life events, showed a significant influence on the level of symptoms as well as on class membership. In the girls’ model, the influence was reduced to that of mothers’ depressive symptoms. Moreover, visual inspection suggests that maternal symptoms showed a stronger influence in boys than girls. Hence, psychosocial influences seem to be more important for boys and neither the influence of mothers’ depressive symptoms nor that of life events can explain why girls show higher symptom severity than boys.

Our results add to the accumulation of inconsistent findings with regard to gender. Some studies showed that the association between psychosocial influences and depressive symptoms were more significant in girls (e.g., Ge et al. 1994; Jenkins and Curwen 2008), whereas others demonstrated no differences between the genders (Johnson et al. 2012). Differences in findings between our study and previous studies may be due to different sample characteristics (size, age, nationality, and vulnerability), measurements (source of information, depression scales, and type of life events), and analytical methods. Furthermore, it is possible that other life events that were not assessed in this study are more salient and stress inducing for girls, for example events that happen with peers (Hankin et al. 2007; Sanchez et al. 2012). Moreover, some former studies have found that girls displayed more functional coping strategies (Jaser et al. 2008) or experienced more social support (Ge et al. 1994; Tiet et al. 1998) than boys. In addition, past research documented significant interactions between cognitive style and psychosocial stressors (Hankin 2009; Rood et al. 2012) as well as between pubertal status and psychosocial factors (Edwards et al. 2011; Ge et al. 2001) in girls. The significance of these factors can of course not be supported by our study. However, to clarify the differences in severity and course of depressive symptoms between girls and boys, it seems to be worthwhile to assess these potential influences in future studies.

Another result deserves closer attention: The “very high-stable” class in girls could not be discriminated from the lowest class by any of our predictors. Although the small sample size of this subgroup may have prevented the detection of a significant influence, we propose that adequate predictors for this subgroup have not yet been identified. Further research is needed to examine the potential ability of other risk factors to increase the probability for membership in the subgroup with the greatest severity of symptoms (e.g., Hankin et al. 2007). This seems to be an issue of particular importance, since there is a high risk for future negative outcomes on psychological health.

Although some questions remain unanswered, our results underline the general importance of psychosocial stressors in predicting different developmental trajectories of depressive symptoms. First, subgroups were faced with different levels of stress. Moreover, the inconsistency in the prediction of class membership suggests that different forms of stressors seem to be more or less relevant for different trajectory subgroups. Stoolmiller et al. (2005) already noted that different vulnerability-stress models might be adequate for different subgroups. Future studies should test this assumption and should assess if subgroups differ in further stressors and/or in their vulnerability to depressive symptoms (e.g., Hankin 2008; Seeds and Dozois 2010). Thus, variations in the reaction to different stressors perhaps can be better understood.

### Strengths and Limitations

Using a large random sample, our study allows new insights into the development of depressive symptoms over time in childhood and adolescence. In general, the age group studied is crucial, because major changes in the symptomatology of depressive symptoms can be expected (Hankin et al. 1998; Rohde et al. 2009). In contrast to the majority of previous studies, we aimed to identify different classes of trajectories for girls and boys separately to present a more differentiated picture. In doing so, we used self-reports of children and adolescents to gain direct insight into their experiences. Furthermore, we analyzed predictive influences through parental reports for a better understanding of the risks for different developmental courses. The use of different sources for the predictor and the outcome variable reduced the risk of a possible reporter bias. Our study thus adds useful information to the results of Dekker et al. (2007). GMM seems to be a powerful and flexible tool and should gain importance for clarifying the existence of different subgroups with varying symptom trajectories (Connell and Frye 2006). In contrast to many other studies, we used the BLRT, which is computationally demanding but currently the most accurate test for model selection (Nylund et al. 2007).

There are also some limitations to this study. Like many other researchers in this field, we used a dimensional measure of depressive symptoms. Therefore, the study focuses mainly on subclinical levels, and the generalization to a clinical population and to depressive disorders is not possible. Because a shortened scale of the YSR was used, it was not possible to compute the proportion of children who reached cut-offs for the clinical or borderline range of depressive symptoms. Furthermore, our comparisons of gender differences are based solely on visual inspection. Without any explicit statistical test of these apparent differences, the results must be interpreted with caution. Some important questions remain unanswered: our predictors neither clarified why girls showed greater severity of symptoms nor did they differentiate the “very high-stable” class in girls. Moreover, the fact that the sample over-represents higher SES levels may have influenced our results in several aspects and could be responsible for differences in results compared with earlier studies. Furthermore, several changes in our predictive variables may have happened during the four-year period with significant impact on the course of depressive symptoms in children and adolescents, for example, a shift in maternal depressive symptoms or the occurrence of new life events.

### Conclusion and Future Research

In general, our findings are in concordance with the majority of previous studies and show compelling evidence that the course of depressive symptoms can demonstrate different, in part chronic, developments. Only a minority of children and adolescents with depressive symptoms are affected by (very) high symptom levels. The large majority undergoes childhood and adolescence with mild symptomatology and probably low impairment of quality of life. However, even those who start with low or moderate levels can show a critical development of symptoms in which the course in girls is more alarming than for boys. Besides, past research has demonstrated that not only severe clinical depression but also subclinical forms of depressive symptoms can interfere with the socio-emotional development and well-being of children and adolescents (Fergusson et al. 2005; Johnson et al. 2009; Pine et al. 1999; Rohde et al. 2009). Our results have several implications for prevention. Children and adolescents who follow different developmental courses require different forms and amounts of social support. Furthermore, the special needs of girls and boys should be addressed. The knowledge of risk factors should further enhance the development and choice of adequate strategies of identification and prevention (Compas et al. 2009; Horowitz and Garber 2006; Yerkey and Wildman 2004). In particular, children whose mothers display a high level of depressive symptoms or boys who suffer from adverse life events are in special need of attention. To gain a greater insight into the gender specific influences on the course of depressive symptoms in childhood and adolescence, future research should analyze the contribution of additional risk or resilience factors, for example, attributional styles (Carter and Garber 2011; Garber et al. 2002), coping styles (Hankin 2009), or social support (Rueger and Malecki 2011; Tiet et al. 1998).

## Footnotes

1 Because it was not possible to revert to standardized norms, depressive symptoms were grouped into four levels using the mean level (*M* = 1.66) and standard deviation (*SD* = 0.58) of symptoms at the first measurement as reference.
